# Adjustment strategies adopted by higher education students during COVID-19 pandemic: focus group discussions

**DOI:** 10.1192/j.eurpsy.2023.1646

**Published:** 2023-07-19

**Authors:** C. Laranjeira, M. Dixe, Z. Charepe, A. Querido

**Affiliations:** 1 School of Health Sciences; 2 ciTechCare, Polytechnic of Leiria, Leiria; 3 Institute of Health Sciences (ICS), Universidade Católica Portuguesa, Lisboa, Portugal

## Abstract

**Introduction:**

With the closure of higher education institutions during the COVID-19 sanitary crisis, students have experienced problems such as interruptions to their education, loss of peer support networks, and mental health issues.

**Objectives:**

This study aimed to explore adjustment patterns used by students to overcome the impact of the COVID-19 pandemic.

**Methods:**

A qualitative descriptive study was developed by carrying out Focus Group Discussions (FGDs). Portuguese students enrolled in education levels above high school, including undergraduate and graduate programs, were considered eligible. Participants were recruited using convenience sampling. Each FGD took approximately 60–90 min.

**Results:**

Twelve students were participated in 2 FGDs, each one with 6 participants. Mostly were undergraduate students (Bachelor’s degree), in the field of health area. The thematic analysis revealed three main themes. The first theme was related to the personal sphere and included most of adjustment strategies used, namely: pandemic as a “window of opportunity” to be involved in new academic and professional projects; work-life balance by organizing and separating work from private life; self-care through the adoption of healthy lifestyles; being compassionate with others and compliance with sanitary measures. In the social sphere, students evoked new ways of communicating via digital networking to compensate for the lack of physical proximity and stay in safe contact with friends and relatives. In the contextual sphere, students talked about the importance of adapting the “teaching/learning” environment. Tailored teaching support was a significant strategy, especially in maintaining their motivation.

**Conclusions:**

Several strategies were pointed by students to stay mentally healthy and mitigate delayed-onset post-traumatic stress disorder during the COVID-19 pandemic. Besides, positive coping and hope should be integrated into the standard training of students across all study areas.

**Disclosure of Interest:**

None Declared

